# Supplementary material for: Endogenous Neuropeptide Nocistatin Is a Direct Agonist of Acid-Sensing Ion Channels (ASIC1, ASIC2 and ASIC3)
Source: Biomolecules. 2019 Aug 22;9(9):401. doi: 10.3390/biom9090401 (PMC6769468; doi:10.3390/biom9090401)
Supplement: Supplementary file 1 [file biomolecules-09-00401-s001.pdf]

Supplementary Materials

1.1. Precursor protein Q62923 (PNOC_RAT) includes only one activator of ASICs

Both neuropeptides nociceptin and orphanin expressed with nocistatin in the same precursor show no effect (neither activating nor inhibitory) onto ASIC1a when they were applied under the same conditions as nocistatin (**Figure S1**).

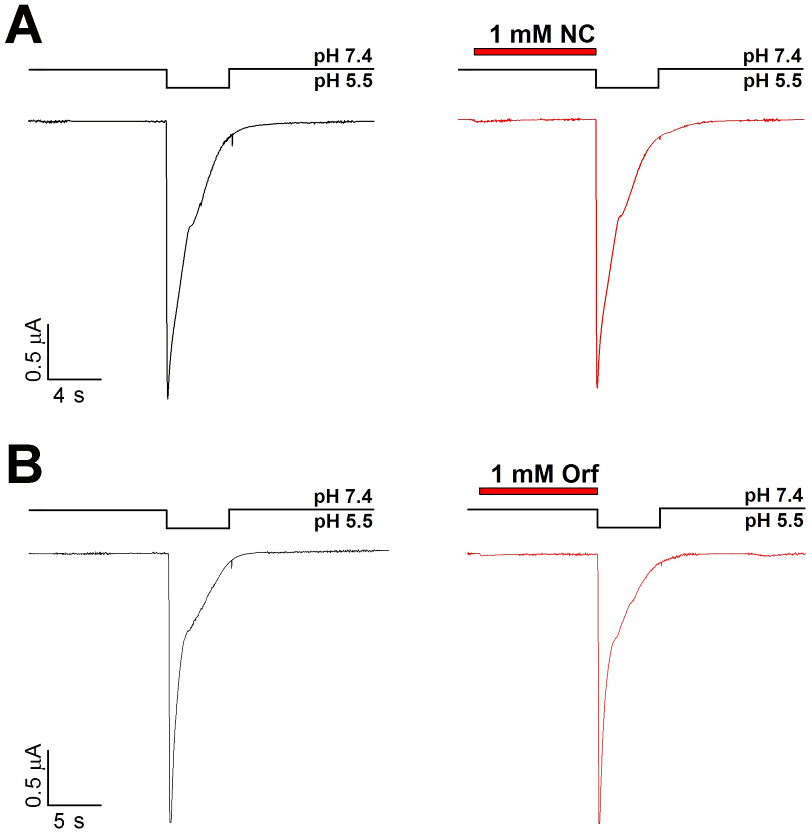

Figure S1. The lack of ASIC1a respond to the application of neuropeptides nociceptin (NC) [PNOC_RAT 135–151] (**A**) and orphanin (Orf) [PNOC_RAT 154–170] (**B**). Currents were measured at a holding external bath pH 7.4 and were induced by a pH drop to 5.5. The left and right panel current traces were obtained on the same cell (n = 4).

1.2. The activating effectiveness of nocistatin on integrated ASIC1a current

The current traces presented in Figure 2 have several shape transformations in depends on nocistatin concentration applied. Therefore, a recalculation of the activating curve (**Figure 2C**) and the inhibitory curve (**Figure 2D, blue and green lines**) was done to estimate effects of nocistatin on the integral current (area under the current trace) (**Figure S2**). The usual logistic equation F_1 failed to perfect fit this curve (dashed line in **Figure S2A).** Calculated $EC_{50}1$ and $EC_{50}2$ values (by equation F_2) did not change significantly (0.27 ± 0.05 and 0.13 ± 0.05 mM, respectively, for integral current (**Figure S2A**) *vs.* 0.247 ± 0.025 and 0.14 ± 0.07 mM for amplitude (**Figure 2C**)), whereas a significant decrease in the Hill coefficient (n_H) occurred (4.8 ± 0.7 for integral current *vs.* 7.44 ± 0.95 for amplitude).

In the case of inhibition effect for nocistatin (**Figure S2B**) the dose-dependence curve of the integral current was fitted much worse by equation F_3 (stand out points 0.25 mM and 0.5 mM concentrations) than in **Figure 2D**. The calculated IC_{50} and n_H values (0.15 ± 0.02 mM and 2.9 ± 0.4) took an intermediate position between values calculated for the amplitude of 1st and 2nd component of the current (see the main text, **Figure 2D**). Thus, we can assume that the analysis of a total measured current is less correct than one for 1st and 2nd current components separately. The summation of the total current values (the first respond to NS + the second respond to pH on (**Figure 2A**) at each point along the abscissa axis in **Figure S2B** gave a log-normal distribution, which was fitted by the log-normal equation (green dashed line).

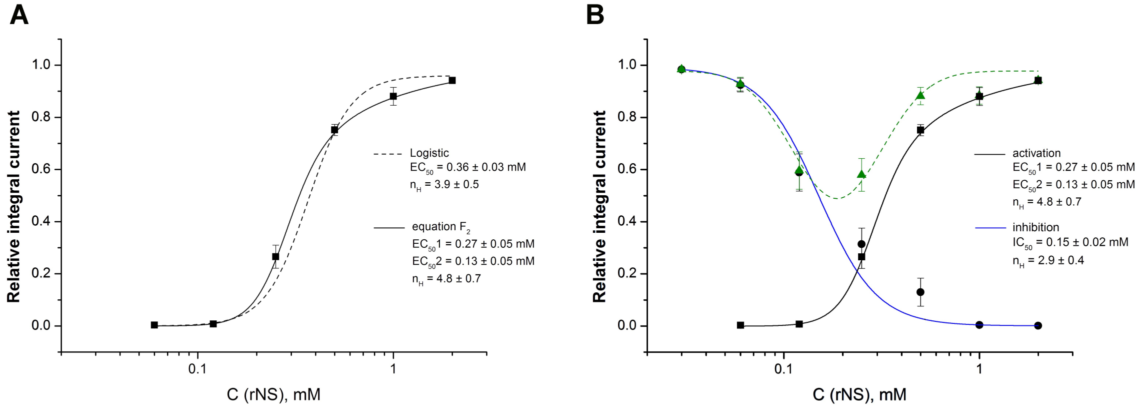

Figure S2. The effect of nocistatin on the integrated current through ASIC1a. (**A**) Dose dependence of ASIC1a activation by rNS fitted by a usual logistic equation $F_1(x)$ (dashed line) and by equation $F_2(x)$ (solid line). The integral current (the area under the current trace) is normalized to the maximum integral current calculated for each oocyte by individual fitting. Data are presented as mean ± SEM; n = 5. (**B**) Dose-dependent integral current decrease (blue line) in response to pH 5.5 stimulus following nocistatin action. The dose response curve of ASIC1a activation by nocistatin (black line) is equal to that presented in

panel A. Green dashed line is a log-normal distribution of summed values from black and blue curves, fitted by the log-normal equation. Data are presented as mean ± SEM; n = 5.
